# Semi-Blind Receivers for Two-Hop MIMO Relay Systems with a Combined TSTF-MSMKron Coding

**DOI:** 10.3390/s23135963

**Published:** 2023-06-27

**Authors:** Pablo H. U. de Pinho, Maria de F. K. B. Couras, Gérard Favier, André L. F. de Almeida, João Paulo J. da Costa

**Affiliations:** 1 University of Brasilia, Federal District, Brasilia 70910-900, Brazil; pablohenriqueifpb@gmail.com (P.H.U.d.P.); kallynna.mary@gmail.com (M.d.F.K.B.C.); joaopaulo.dacosta@hshl.de (J.P.J.d.C.); 2 I3S Laboratory, CNRS, Université Côte d’Azur, 06903 Sophia Antipolis, France; 3 Federal University of Ceara, Fortaleza 60020-181, Ceara, Brazil; andre@gtel.ufc.br; 4 Hamm-Lippstadt University of Applied Sciences, 59063 Hamm, Germany

**Keywords:** generalized Tucker decomposition, MIMO relaying, multiple Kronecker product, semi-blind receivers, TSTF-MSMKron coding

## Abstract

Due to the increase in the number of mobile stations in recent years, cooperative relaying systems have emerged as a promising technique for improving the quality of fifth-generation (5G) wireless networks with an extension of the coverage area. In this paper, we propose a two-hop orthogonal frequency division multiplexing and code-division multiple-access (OFDM-CDMA) multiple-input multiple-output (MIMO) relay system, which combines, both at the source and relay nodes, a tensor space–time–frequency (TSTF) coding with a multiple symbol matrices Kronecker product (MSMKron), called TSTF-MSMKron coding, aiming to increase the diversity gain. It is first established that the signals received at the relay and the destination satisfy generalized Tucker models whose core tensors are the coding tensors. Assuming the coding tensors are known at both nodes, tensor models are exploited to derive two semi-blind receivers, composed of two steps, to jointly estimate symbol matrices and individual channels. Necessary conditions for parameter identifiability with each receiver are established. Extensive Monte Carlo simulation results are provided to show the impact of design parameters on the symbol error rate (SER) performance, using the zero-forcing (ZF) receiver. Next, Monte Carlo simulations illustrate the effectiveness of the proposed TSTF-MSMKron coding and semi-blind receivers, highlighting the benefit of exploiting the new coding to increase the diversity gain.

## 1. Introduction

In recent years, wireless communication systems have experienced great growth in the number of users and new applications such as autonomous vehicles, smart homes, Internet of Things (IoT) and virtual/augmented reality. Compared to fourth-generation (4G) wireless systems, 5G ones offer advantages in terms of data rate, reliability, latency, energy efficiency, and mobility. To fulfill these objectives, 5G needs to operate at high frequency bands, with more base stations in a smaller area, to provide a better reliability and transmission quality to the users [1,2,3].

That explains why in the last few years, cooperative multiple-input multiple-output (MIMO) systems have attracted a lot of attention for 5G mobile networks to increase the transmission coverage area, data rates and performance of wireless communications [4]. Cooperative MIMO systems provide spatial diversity and spatial multiplexing due to the use of multiple antennas to transmit and receive signals at each node of the systems. However, individual channel estimation in a cooperative MIMO system is a fundamental problem to solve, since the reliability of the system greatly depends on the accuracy of channel state information (CSI) in each hop.

During the last two decades, tensor models have been widely used for designing wireless communication systems [5,6]. Tensor-based approaches allow taking different diversities (space, time, frequency, code, polarization, etc.) into account during the system design and developing semi-blind receivers for jointly estimating the channels and symbol matrices under more relaxed conditions than matrix-based methods. Many receivers exploit the two most popular tensor decompositions, namely the Tucker [7] and Parallel Factors Analysis (PARAFAC) [8] models, as in [9,10,11,12,13,14]. However, during the last decade, the design of tensor-based wireless communication systems has led to the development of several new tensor models such as, for instance, the nested PARAFAC [15] and nested Tucker [16] models. See for instance their use in the context of point-to-point MIMO systems [17] and cooperative MIMO systems [18,19,20,21,22].

In the context of cooperative systems, some works are dedicated to the use of a training sequence for estimating the channels in a supervised way, as in [14,23]. Such supervised systems are bandwidth-consuming, which explains the development of semi-blind receivers to jointly estimate the transmitted information symbols and the channels, i.e., without the use of training sequences, such as in the case for the systems briefly introduced below. Many works combine cooperative MIMO systems with different space/time/frequency codings to increase system diversity and obtain better performance in terms of channel and symbol estimation. Among the used codings, one can mention the Khatri–Rao space–time (KRST) coding [18,19,24,25], the multiple Khatri–Rao and Kronecker space–time (MKRST and MKronST) codings [17,26], the tensor space–time (TST) [27,28,29] and tensor–space–time–frequency (TSTF) codings [30]. Depending on the coding chosen for the relay system, different tensor models are obtained for the signals received at the relay and destination nodes. An exploitation of these models makes it possible to derive two families of receivers. One is made up of the most common receivers based on iterative algorithms such as alternating least squares (ALS) or the Levenbergh–Marquardt (LM) method. The other is composed of closed-form algorithms based on singular value decomposition (SVD) calculation, such as Khatri–Rao and Kronecker factorization algorithms, which are denoted KRF and KronF respectively.

In Table 1, the tensor-based MIMO cooperative systems of the above cited references are compared in terms of system type (number of hops), coding, tensor model, and receiver, with the proposed MIMO relay system, which is referenced as “New” in Table 1.

We now briefly comment on the relay systems compared in Table 1 from a historical perspective. First, it is important to note that all these systems consider an amplify-and-forward (AF) protocol at the relays except the system in [26] for which the AF protocol is compared with the decode-and-forward (DF) and estimate-and-forward (EF) ones, showing that the use of these last two protocols allows significantly improving the SER performance at the cost of an additional computational complexity at the relay. From a coding point of view, the Khatri–Rao space–time (KRST) coding was firstly used in [18,19,24] for a two-hop system and then in [25] for a multi-hop system. In [22], KRST coding is combined with a rotation coding matrix for a three-hop system.

The tensor space–time (TST) coding initialy proposed in [27], in the context of point-to-point systems, was used for a two-hop system in [16], leading to a new tensor model called nested Tucker decomposition (TD) and then for a multi-hop system in [21]. In this last reference, a new tensor model called high-order nested Tucker decomposition (HONTD) was introduced. In [28], TST coding is used in a two-hop multi-relay system where the relays directly and sequentially communicate with the destination node. The sequential transmission from the relays to the destination leads to a new coupled nested TD model. In [29], TST coding is combined with a PARAFAC coding structure for a two half-duplex relays system. Two new codings, denoted MKRST and MKronST, were proposed in [26] for a two-hop system, leading to a nested PARAFAC model for the tensor of signals received at destination which is exploited to develop closed-form semi-blind receivers for joint symbol and channel estimation.

An important difference between the systems in Table 1 and the system presented in this paper concerns the a priori information needed to eliminate scaling ambiguities. Thus, our system only requires a priori knowledge of one entry of the symbol matrices, whereas all the systems in Table 1 also require knowledge of one entry or of one row of the channel matrices, which is a much more restrictive assumption.

This paper proposes a new two-hop OFDM-CDMA MIMO relay system which combines a tensor space–time–frequency (TSTF) coding with a multiple Kronecker product of symbol matrices (MSMKron) at the source and relay nodes. This new coding scheme, called TSTF-MSMKron coding, can be viewed as a generalization of the codings proposed in [26,30], with the aim of increasing the diversity gain. It is established that the signals received at the relay and destination nodes satisfy generalized Tucker models whose core tensors are the coding tensors. Assuming the coding tensors are known at both nodes, the multilinear structure of tensor models is exploited to derive two semi-blind receivers for jointly estimating the symbol matrices and individual channels. Necessary conditions for parameter identifiability with each receiver are established. Extensive Monte Carlo simulations illustrate the effectiveness of the proposed TSTF-MSMKron coding and semi-blind receivers. Note that our two-hop MIMO relay system differs mainly from the systems compared in Table 1 by the proposed TSTF-MSMMKron coding scheme which induces a greater diversity gain than the codings used by the systems referenced in Table 1. Another important difference lies in the consideration of frequency-dependent channels, i.e., three-dimensional channels. These assumptions lead to received signal tensors at the relay and the destination that satisfy generalized Tucker models whose essential uniqueness is ensured by the a priori knowledge of coding tensors. Scalar ambiguities can be eliminated assuming the knowledge of only one symbol per each symbol matrix. Exploiting the tensor models of received signals allows developing two types of semi-blind receivers for estimating the information symbols and the individual channels: one is iterative based on the Bi-ALS algorithm to estimate each individual channel and the Kronecker product of symbol matrices, combined with the KronF method to separate the symbol matrices, while the other one is closed form and based on the THOSVD algorithm [31], which allows simultaneously estimating each individual channel and symbol matrix. Note that unlike almost all relay systems existing in the literature which use the AF protocol, the proposed two-hop system uses the DF protocol at the relay, which greatly facilitates its generalization to the multi-hop case.

The main contributions of the paper can be summarized as follows:

- A new two-hop OFDM-CDMA system that combines a TSTF coding with a multiple Kronecker product of symbol matrices (MSMKron) at the source and relay nodes is proposed.
- It is established that the tensor of signals received at each hop satisfies a generalized Tucker model.
- By exploiting the tensor model of the signals received at the relay and destination nodes, two semi-blind receivers are derived to jointly estimate the individual source–relay and relay–destination channels and transmitted symbols.
- System model uniqueness and parameter identifiability conditions for each proposed receiver are analyzed.
- The performance of the TSTF-MSMKron coding and the impact of design parameters on the symbol error rate (SER) are first evaluated using the zero-forcing (ZF) receiver, i.e., under the assumption of a perfect channel knowledge, by means of extensive Monte Carlo simulations. Then, the proposed semi-blind receivers are compared for symbol and channel estimation.

The rest of the paper is organized as follows. Section 2 presents tensor preliminaries. Section 3 first describes the system model, presenting the TSTF-MSMKron coding and the signals received at the relay and destination. These signals form two tensors that satisfy generalized Tucker decompositions. In Section 4, two semi-blind receivers are proposed to jointly estimate the symbol matrices and channels. Necessary conditions for parameter identifiability are derived for each receiver. In Section 6, extensive Monte Carlo simulation results are provided to illustrate the effectiveness of the proposed two-hop relay system. Section 7 concludes the paper.

**Notation:** scalars, column vectors, matrices, and tensors are denoted by lowercase, boldface lowercase, boldface uppercase and boldface calligraphic letters, e.g., *x*, x, **X**, and X, respectively. The transpose, complex conjugate, complex conjugate transpose, and Moore–Penrose pseudo-inverse of X are represented by $X^{T},X^{*}$, $X^{H}$ and $X^{†}$, respectively. We denote by $x_{i,j}$ the (i,j) element and by $X_{i.}$ (resp. $X_{.j}$) the *i*th row (resp. *j*th column) of $X\in C^{I\times J}$. The ($i_{1}$, …, $i_{N}$) element of the *N*-order tensor $X\in C^{I_{1}\times \ldots \times I_{N}}$ will be written $x_{i_{1},\ldots ,i_{N}}$. $I_{R}$ and $I_{N,R}$ represent the identity matrix of size R×R and the identity tensor of *N*-order and size R×R×….×R, respectively. $\hat{X}$ denotes an estimate of X and $\hat{\hat{X}}$ represents the matrix $\hat{X}$ after ambiguities suppression.

$X_{I_{1}\times I_{2}I_{3}}$ represents an unfolding of the third-order tensor $X\in C^{I_{1}\times I_{2}\times I_{3}}$ of dimension $I_{1}\times I_{2}I_{3}$. The vec and unvec operators are defined by $x_{I_{2}I_{3}I_{1}}=\mathrm{vec}\left(X_{I_{1}\times I_{2}I_{3}}\right)\in C^{I_{2}I_{3}I_{1}}↔X_{I_{1}\times I_{2}I_{3}}=\mathrm{unvec}\left(x_{I_{2}I_{3}I_{1}}\right)$. By slicing the third-order tensor X along each mode, we obtain three types of matrix slices called horizontal, lateral, and frontal slices, which are denoted, respectively, as follows:

$$X_{i_{1}..}\in C^{I_{2}\times I_{3}},\;X_{.i_{2}.}\in C^{I_{3}\times I_{1}}\mathrm{and}X_{..i_{3}}\in C^{I_{1}\times I_{2}},$$

with $i_{1}\in \left[1,I_{1}\right]$, $i_{2}\in \left[1,I_{2}\right]$ and $i_{3}\in \left[1,I_{3}\right]$. The Kronecker, Khatri–Rao, and outer products are denoted by ⊗, ⋄, and ∘, respectively. The operator bdiag(.) forms a block-diagonal matrix from its matrix arguments, with $\mathrm{bdiag}\left(X_{..k}\right)≜\mathrm{bdiag}\left(X_{..1},\ldots ,X_{..K}\right)\in C^{KI\times KJ}$, where $X_{..k}\in C^{I\times J}$ is the *k*th frontal slice of $X\in C^{I\times J\times K}$.

All acronyms used in the paper are summarized after Section 7.

## 2. Tensor Preliminaries

The mode-*n* product between a tensor $G\in C^{R_{1}\times \ldots \times R_{n-1}\times R_{n}\times R_{n+1}\times \ldots \times R_{N}}$ and a matrix $A\in C^{I_{n}\times R_{n}}$, denoted by $G\times_{n}A$, gives an *N*-order tensor X of size $R_{1}\times \ldots \times R_{n-1}\times I_{n}\times R_{n+1}\times \ldots \times R_{N}$, which is defined by:

(1) $$x_{r_{1},\ldots ,r_{n-1},i_{n},r_{n+1},\ldots ,r_{N}}=\sum_{r_{n}=1}^{R_{n}}g_{r_{1},\ldots ,r_{n-1},r_{n},r_{n+1},\ldots ,r_{N}}a_{i_{n},r_{n}}.$$

The mode-*n* product between two tensors $G\in C^{R_{1}\times \ldots \times R_{n-1}\times R_{n}\times R_{n+1}\times \ldots \times R_{N_{1}}\times I_{N_{1}+1}\times \ldots \times I_{N}}$ and $A\in C^{I_{n}\times R_{n}\times I_{N_{1}+1}\times \ldots \times I_{N}}$ is denoted by $G\times_{n}A$, with $n\in [1,N_{1}]$. This product gives an *N*-order tensor $X\in C^{R_{1}\times \ldots \times R_{n-1}\times I_{n}\times R_{n+1}\times \ldots \times R_{N_{1}}\times I_{N_{1}+1}\times \ldots \times I_{N}}$, which is defined as [30]:

(2) $$x_{r_{1},\ldots ,r_{n-1},i_{n},r_{n+1},\ldots ,r_{N_{1}},i_{N_{1}+1},\ldots ,i_{N}}=\sum_{r_{n}=1}^{R_{n}}g_{r_{1},\ldots ,r_{n-1},r_{n},r_{n+1},\ldots ,r_{N_{1}},i_{N_{1}+1},\ldots ,i_{N}}a_{i_{n},r_{n},i_{N_{1}+1},\ldots ,i_{N}}.$$

The sum is over the second index of the tensor A, as for the mode-*n* product (1) between a tensor and a matrix. For example, consider the third-order tensors $G\in C^{R_{1}\times I_{2}\times I_{3}}$ and $A\in C^{I_{1}\times R_{1}\times I_{3}}$. The mode-1 product $X=G\times_{1}A$ is given by:

(3) $$x_{i_{1},i_{2},i_{3}}=\sum_{r_{1}}^{R_{1}}g_{r_{1},i_{2},i_{3}}a_{i_{1},r_{1},i_{3}}.$$

We now introduce the notion of a generalized Tucker-($N_{1},N$) model for an *N*-order tensor $X\in C^{I_{1}\times \ldots \times I_{N}}$, with $N_{1}<N$, which is defined as [32,33]:

(4) $$x_{i_{1},..,i_{N}}=\sum_{r_{1}=1}^{R_{1}}\ldots \sum_{r_{N_{1}}=1}^{R_{N_{1}}}g_{r_{1},\ldots ,r_{N_{1}},i_{N_{1}+1},\ldots ,i_{N}}\prod_{n=1}^{N_{1}}a_{i_{n},r_{n},S_{n}}^{\left(n\right)}.$$

where $S_{n}$ is an ordered subset of the set $\left\{i_{N_{1}+1},\ldots ,i_{N}\right\}$. This model can be written in terms of mode-*n* products as:

(5) $$X=G\times_{n=1}^{N_{1}}A^{\left(n\right)},$$

where $G\in C^{R_{1}\times \ldots \times R_{N_{1}}\times I_{N_{1}+1}\times \ldots \times I_{N}}$ is the core tensor, and $A^{\left(n\right)}\in C^{I_{n}\times R_{n}\times J_{n}}$ are tensor factors for $n\in [1,N_{1}]$, where $J_{n}$ is a subset of $\left\{I_{N_{1}+1},\ldots ,I_{N}\right\}$. For example, let us consider two factors, where the first one is a third-order tensor $A^{\left(1\right)}\in C^{I_{1}\times R_{1}\times I_{3}}$ and the second one is a matrix $A^{\left(2\right)}\in C^{I_{2}\times R_{2}}$. A generalized Tucker-(2,4) model is given by:

(6) $$X=G\times_{1}A^{\left(1\right)}\times_{2}A^{\left(2\right)}\in C^{I_{1}\times I_{2}\times I_{3}\times I_{4}},$$

where $G\in C^{R_{1}\times R_{2}\times I_{3}\times I_{4}}$. In scalar form, Equation (6) can be written as:

(7) $$x_{i_{1},i_{2},i_{3},i_{4}}=\sum_{r_{1}=1}^{R_{1}}\sum_{r_{2}=1}^{R_{2}}g_{r_{1},r_{2},i_{3},i_{4}}a_{i_{1},r_{1},i_{3}}^{\left(1\right)}a_{i_{2},r_{2}}^{\left(2\right)}.$$

## 3. System Model

### 3.1. Presentation of the Proposed Two-Hop System

**Figure 1:**
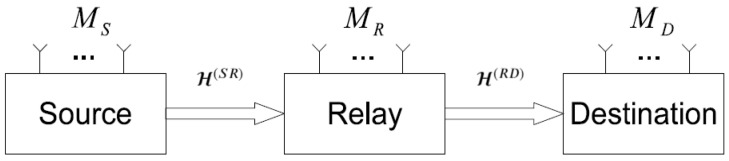
Block diagram of the two-hop MIMO relay system.

Consider a two-hop MIMO OFDM-CDMA system, as illustrated in Figure 1. This system is equipped with $M_{S}$, $M_{R}$ and $M_{D}$ antennas at the source, relay and destination nodes, respectively. The source–relay ($H^{\left(SR\right)}\in C^{M_{R}\times M_{S}\times F}$) and relay–destination ($H^{\left(RD\right)}\in C^{M_{D}\times M_{R}\times F}$) channels are assumed to be flat Rayleigh fading, which is represented by third-order tensors whose coefficients are zero-mean circularly symmetric complex Gaussian i.i.d. (independent and identically distributed) random variables that are constant during at least *P* transmission blocks.

The decode-and-forward (DF) protocol is considered at the relay, and the transmission occurs in two hops. During the first one, the coded symbols are transmitted by the source to the relay via the channel $H^{\left(SR\right)}$ and decoded at the relay. During the second one, the estimated symbols are re-encoded and then re-transmitted by the relay to the destination via the channel $H^{\left(RD\right)}$. Each symbol matrix $S^{\left(l\right)}=\left[s_{n_{l},r_{l}}^{\left(l\right)}\right]\in C^{N_{l}\times R_{l}}$, with $r_{l}\in \left[1,R_{l}\right],n_{l}\in \left[1,N_{l}\right]$, for l∈[1,L], is composed of $R_{l}$ data streams, each one containing $N_{l}$ information symbols. The transmission protocol is detailed in the next section which defines the TSTF-MSMKron coding. Then, in Section 3.3 and Section 3.4, the tensors of signals received at the relay and the destination will be described, respectively.

### 3.2. TSTF-MSMKron Coding

In the proposed relay system, the coding at the source node is composed of two steps. During the first one, a multiple Kronecker product of *L* symbol matrices is calculated as:

(8) $$S={⊗}_{l=1}^{L}S^{\left(l\right)}≜S^{\left(1\right)}⊗\ldots ⊗S^{\left(L\right)}\in C^{N\times R},$$

where $N=\prod_{l=1}^{L}N_{l}$, and $R=\prod_{l=1}^{L}R_{l}$. The scalar form of (8) is:

(9) $$s_{n,r}=\prod_{l=1}^{L}s_{n_{l},r_{l}}^{\left(l\right)},\;\;\;n\in \left[1,N\right]\;,\;\;\;r\in \left[1,R\right],$$

with $n=n_{L}^{\left(L\right)}+\left(n_{L-1}^{\left(L-1\right)}-1\right)N_{L}+\cdots +\left(n_{1}^{\left(1\right)}-1\right)\prod_{l=2}^{L}N_{l}$, and $r=r_{L}^{\left(L\right)}+\left(r_{L-1}^{\left(L-1\right)}-1\right)R_{L}+\cdots +\left(r_{1}^{\left(1\right)}-1\right)\prod_{l=2}^{L}R_{l}$, where $n_{l}^{\left(l\right)}\in \left[1,\;N_{l}\right]$ and $r_{l}^{\left(l\right)}\in \left[1,\;R_{l}\right]$ denote the indices $n_{l}$ and $r_{l}$ in $s_{n_{l},r_{l}}^{\left(l\right)}$. This operation, called MSMKron coding, corresponds to a simplified version of the MKronST coding [26] without a known precoding matrix. This coding induces time and code spreadings of each symbol $s_{n_{l},r_{l}}^{\left(l\right)}$ due to the multiple Kronecker product of the symbol matrix $S^{\left(l\right)}$ with the other matrices $S^{\left(l^{\prime }\right)}$, $l^{\prime }=1,\cdots ,L$ and $l^{\prime }\neq l$.

The transmission being composed of *P* time-slots means each symbol $s_{n_{l},r_{l}}^{\left(l\right)}$ is repeated $P\left(\prod_{\begin{array}{c} l^{\prime }=1\\ l^{\prime }\neq l \end{array}}^{L}N_{l^{\prime }}\right)(\prod_{\begin{array}{c} l^{\prime }=1\\ l^{\prime }\neq l \end{array}}^{L}R_{l^{\prime }})$ times, which implies an increase of time and code diversities when increasing the dimensions $N_{l}$ and $R_{l}$, respectively.

During the second step, the MSMKron coding is combined with a tensor space–time–frequency (TSTF) coding [30] carried out by means of the (L+3)-order tensor $G^{\left(S\right)}\in C^{M_{S}\times R_{1}\times \ldots \times R_{L}\times F\times P}$ in such a way that the tensor of signals coded at the source satisfies an (L+3)-order Tucker model given by:

(10) $$V^{\left(S\right)}=G^{\left(S\right)}\times_{1}I_{M_{S}}\times_{2}S^{\left(1\right)}\times_{3}\ldots \times_{L+1}S^{\left(L\right)}\times_{L+2}I_{F}\times_{L+3}I_{P}\in C^{M_{S}\times N_{1}\times \ldots \times N_{L}\times F\times P}.$$

Note that the core tensor of this decomposition is the coding tensor $G^{\left(S\right)}$. In scalar notation, the coded signals transmitted by the $m_{S}^{th}$ antenna at the source, using the $f^{th}$ subcarrier, during the $p^{th}$ time slot are given by:

(11) $$v_{m_{S},n_{1},...,n_{L},f,p}^{\left(S\right)}=\sum_{r_{1}=1}^{R_{1}}...\sum_{r_{L}=1}^{R_{L}}g_{m_{S},r_{1},...,r_{L},f,p}^{\left(S\right)}\prod_{l=1}^{L}s_{n_{l},r_{l}}^{\left(l\right)}$$

where $m_{S}\in \left[1,M_{S}\right],f\in \left[1,F\right],p\in \left[1,P\right]$. The TSTF-MSMKron coding increases space–time–frequency diversity, as will be illustrated in the simulations.

### 3.3. Tensor of Signals Received at the Relay

In the noise-free case and assuming a flat Rayleigh fading propagation channel, the signal $x_{m_{R},n_{1},\ldots ,n_{L},f,p}^{\left(SR\right)}$ received at the $m_{R}^{th}$ antenna of the relay, during the $n_{l}^{th}$ symbol period of the $p^{th}$ block and associated with the $f^{th}$ subcarrier, is given by:

(12) $$x_{m_{R},n_{1},\ldots ,n_{L},f,p}^{\left(SR\right)}=\sum_{m_{S}=1}^{M_{S}}h_{m_{R},m_{S},f}^{\left(SR\right)}v_{m_{S},n_{1},\ldots ,n_{L},f,p}^{\left(S\right)}$$

where $m_{R}\in \left[1,M_{R}\right]$ and $h_{m_{R},m_{S},f}^{\left(SR\right)}$ is an entry of the channel $H^{\left(SR\right)}\in C^{M_{R}\times M_{S}\times F}$. In terms of mode-*n* products, we have:

(13) $$X^{\left(SR\right)}=V^{\left(S\right)}\times_{1}H^{\left(SR\right)}\in C^{M_{R}\times N_{1}\times \ldots \times N_{L}\times F\times P}.$$

Note that the transmission via channel $H^{\left(SR\right)}$ can be interpreted as a mode-1 linear transformation applied to the tensor $V^{\left(S\right)}$ of coded signals. Substituting (11) into (12) gives the signal received at the relay written in scalar form as:

(14) $$x_{m_{R},n_{1},\ldots ,n_{L},f,p}^{\left(SR\right)}=\sum_{m_{S}=1}^{M_{S}}\sum_{r_{1}=1}^{R_{1}}\ldots \sum_{r_{L}=1}^{R_{L}}g_{m_{S},r_{1},\ldots ,r_{L},f,p}^{\left(S\right)}h_{m_{R},m_{S},f}^{\left(SR\right)}\prod_{l=1}^{L}s_{n_{l},r_{l}}^{\left(l\right)}.$$

The signals received at the relay form the tensor $X^{\left(SR\right)}$ that satisfies a generalized Tucker-(L+1,L+3) model given by:

(15) $$X^{\left(SR\right)}=G^{\left(S\right)}\times_{1}H^{\left(SR\right)}\times_{2}S^{\left(1\right)}\times_{3}\ldots \times_{L+1}S^{\left(L\right)}\times_{L+2}I_{F}\times_{L+3}I_{P},$$

where $S^{\left(l\right)}$ represents the symbol matrices encoded by the TSTF-MSMKron coding for l∈[1,L], and $G^{\left(S\right)}$ is the core tensor of the Tucker model. As is well known, knowledge of the core tensor implies the uniqueness of this model. Combining modes 2 to L+1 of tensors $G^{\left(S\right)}$ and $X^{\left(SR\right)}$ results in contracted forms $G_{c}^{\left(S\right)}\in C^{M_{S}\times R\times F\times P}$ and $X_{c}^{\left(SR\right)}\in C^{M_{R}\times N\times F\times P}$, and Equation (15) can be rewritten as:

(16) $$X_{c}^{\left(SR\right)}=G_{c}^{\left(S\right)}\times_{1}H^{\left(SR\right)}\times_{2}S\times_{3}I_{F}\times_{4}I_{P}.$$

From the Tucker model (16), it is easy to deduce the following matrix unfoldings of the tensor $X^{\left(SR\right)}$:

(17) $$X_{FPN\times M_{R}}^{\left(SR\right)}=\left(I_{FP}⊗S\right)G_{FPR\times FM_{S}}^{\left(S\right)}H_{FM_{S}\times M_{R}}^{\left(SR\right)}\in C^{FPN\times M_{R}},$$

(18) $$X_{PFM_{R}\times N}^{\left(SR\right)}=\left(I_{P}⊗\mathrm{bdiag}\left(H_{..f}^{\left(SR\right)}\right)\right)G_{PFM_{S}\times R}^{\left(S\right)}S^{T}\in C^{PFM_{R}\times N},$$

(19) $$X_{M_{R}N\times FP}^{\left(SR\right)}=\left(H_{M_{R}\times FM_{S}}^{\left(SR\right)}⊗S\right)G_{FM_{S}R\times FP}^{\left(S\right)}\in C^{M_{R}N\times FP},$$

with $H_{..f}^{\left(SR\right)}\in C^{M_{R}\times M_{S}}$ and bdiag(.) previously defined in the notation. Note that the identity matrix $I_{FP}\in R^{FP\times FP}$ in (17) is associated with FP repetitions of the symbol matrices inducing time-frequency diversity for the system.

The block structure of the matrix unfoldings $G_{FPR\times FM_{S}}^{\left(S\right)}$ and $G_{FM_{S}R\times FP}^{\left(S\right)}$ in Equations (17) and (19), respectively, is defined as follows:

(20) $$G_{FPR\times FM_{S}}^{\left(S\right)}=\mathrm{bdiag}\left[\begin{array}{ccc} \mathrm{vec}(G_{1..f.}^{\left(S\right)}) & \ldots & \mathrm{vec}(G_{M_{S}..f.}^{\left(S\right)}) \end{array}\right]=\mathrm{bdiag}\left({\left[G_{PR\times M_{S}}^{\left(S\right)}\right]}_{f}\right),$$

(21) $$G_{FM_{S}R\times FP}^{\left(S\right)}=\mathrm{bdiag}\left[\begin{array}{ccc} \mathrm{vec}(G_{\ldots f1}^{\left(S\right)}) & \ldots & \mathrm{vec}(G_{\ldots fP}^{\left(S\right)}) \end{array}\right]=\mathrm{bdiag}\left({\left[G_{M_{S}R\times P}^{\left(S\right)}\right]}_{f}\right).$$

$G_{FPR\times FM_{S}}^{\left(S\right)}$ in (20) is a block-diagonal matrix, formed of *F* diagonal blocks of dimension $PR\times M_{S}$, each block being formed of $M_{S}$ column vectors corresponding to a vectorized form of the tensor slice $G_{m_{S}..f.}^{\left(S\right)}$ of size $R_{1}\times \ldots \times R_{L}\times P$, for $m_{S}\in \left[1,M_{S}\right]$, such that $\mathrm{vec}\left(G_{m_{S}..f.}^{\left(S\right)}\right)\in C^{PR}$. Similarly, $G_{FM_{S}R\times FP}^{\left(S\right)}$ in (21) is a block-diagonal matrix whose diagonal blocks are of dimension $M_{S}R\times P$, with $\mathrm{vec}\left(G_{\ldots fp}^{\left(S\right)}\right)\in C^{M_{S}R}$.

To illustrate the matrix unfolding (21), consider the case where $R=P=M_{S}=F=2$, leading to the following matrix:

(22) $$G_{FM_{S}R\times FP}^{\left(S\right)}=\left[\begin{array}{cc} \begin{array}{cc} g_{1111}^{\left(S\right)} & g_{1112}^{\left(S\right)}\\ g_{2111}^{\left(S\right)} & g_{2112}^{\left(S\right)}\\ g_{1211}^{\left(S\right)} & g_{1212}^{\left(S\right)}\\ g_{2211}^{\left(S\right)} & g_{2212}^{\left(S\right)} \end{array} & \begin{array}{cc} 0 & 0\\ 0 & 0\\ 0 & 0\\ 0 & 0 \end{array}\\ \begin{array}{cc} 0 & 0\\ 0 & 0\\ 0 & 0\\ 0 & 0 \end{array} & \begin{array}{cc} g_{1121}^{\left(S\right)} & g_{1122}^{\left(S\right)}\\ g_{2121}^{\left(S\right)} & g_{2122}^{\left(S\right)}\\ g_{1221}^{\left(S\right)} & g_{1222}^{\left(S\right)}\\ g_{2221}^{\left(S\right)} & g_{2222}^{\left(S\right)} \end{array} \end{array}\right].$$

### 3.4. Tensor of Signals Received at the Destination

With the DF protocol, the symbols received at the relay are first decoded by means of one of the receivers described in Section 4, leading to the estimated symbol matrices ${\hat{\hat{S}}}^{\left(l\right)}$, which are also written as $S_{R}^{\left(l\right)}$. The estimated symbols are then re-encoded at the relay using a TSTF-MSMKron coding, with the tensor coding $G^{\left(R\right)}\in C^{M_{R}\times R_{1}\times ...\times R_{L}\times F\times P}$. The re-encoded signals are transmitted by the relay to the destination via the channel $H^{\left(RD\right)}\in C^{M_{D}\times M_{R}\times F}$. The signals received at the destination are similar to the signals received at the relay, defined by Equations (14) and (15), with the following correspondences:

(23) $$\left(G^{\left(S\right)},H^{\left(SR\right)},S^{\left(l\right)}\right)↔\left(G^{\left(R\right)},H^{\left(RD\right)},S_{R}^{\left(l\right)}\right),$$

(24) $$\left(M_{R},M_{S}\right)↔\left(M_{D},M_{R}\right),$$

Similar to (14), in the noiseless case, the signal received at the $m_{D}^{th}$ antenna of the destination node, during the $n_{l}^{th}$ symbol period of the $p^{th}$ time block and associated with the $f^{th}$ subcarrier, is given by:

(25) $$x_{m_{D},n_{1},...,n_{L},f,p}^{\left(RD\right)}=\sum_{m_{R}=1}^{M_{R}}\sum_{r_{1}=1}^{R_{1}}...\sum_{r_{L}=1}^{R_{L}}g_{m_{R},r_{1},...,r_{L},f,p}^{\left(R\right)}h_{m_{D},m_{R},f}^{\left(RD\right)}\prod_{l=1}^{L}{\left[s_{R}^{\left(l\right)}\right]}_{n_{l},r_{l}},$$

and the generalized Tucker-(L+1, L+3) model (15) becomes:

(26) $$X^{\left(RD\right)}=G^{\left(R\right)}\times_{1}H^{\left(RD\right)}\times_{2}S_{R}^{\left(1\right)}\times_{3}...\times_{L+1}S_{R}^{\left(L\right)}\times_{L+2}I_{F}\times_{L+3}I_{P},$$

where $X^{\left(RD\right)}\in C^{M_{D}\times N_{1}\times ...\times N_{L}\times F\times P}$. Matrix unfoldings of this tensor can be deduced from (17)–(19) using the correspondences (23) and (24) with $G_{PFM_{R}\times R}^{\left(R\right)}$, $G_{FPR\times FM_{R}}^{\left(R\right)}$ and $G_{FM_{R}R\times FP}^{\left(R\right)}$ instead of $G_{PFM_{S}\times R}^{\left(S\right)}$, $G_{FPR\times FM_{S}}^{\left(S\right)}$ and $G_{FM_{S}R\times FP}^{\left(S\right)}$, respectively.

**Figure 2:**
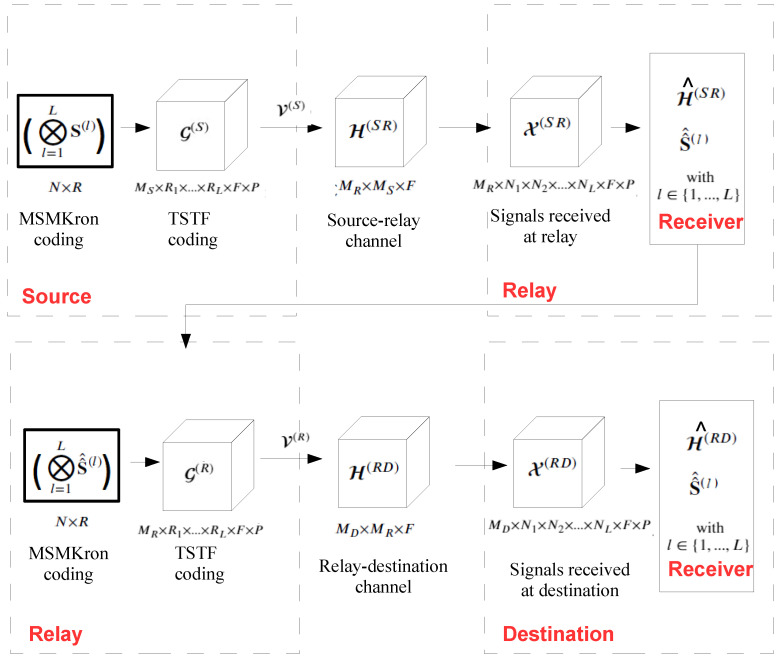
Block diagram of the proposed two-hop MIMO OFDM-CDMA communication system.

The proposed OFDM-CDMA relaying system is illustrated by means of the block diagram in Figure 2.

The system design parameters and the definitions of the system matrices and tensors are summarized in Table 2 and Table 3, respectively.

## 4. Semi-Blind Receivers

In this section, two semi-blind receivers are proposed to estimate the channel tensors and symbol matrices at the relay and destination nodes. We assume that the coding tensors $G^{\left(S\right)}$ and $G^{\left(R\right)}$ are known. We also assume that one symbol of each symbol matrix is known to eliminate scalar ambiguities. The symbol matrices and the channel tensor $H^{\left(SR\right)}$ are estimated at the relay, while the symbol matrices and the channel tensor $H^{\left(RD\right)}$ are estimated at the destination. The proposed receivers are detailed for the relay. The same receivers can be derived for the destination, using the correspondences (23) and (24). The first one is based on the alternating least squares (ALS) algorithm to estimate the channel and the Kronecker product of symbol matrices, which is followed by the Kronecker factorization (KronF) method to separate the symbol matrices, while the second one is a closed-form solution allowing to jointly estimate the channel and the symbol matrices by means of the truncated higher-order singular value decomposition (THOSVD) algorithm.

### 4.1. Bi-ALS-KronF Receiver

In the first step, the bi-alternating least squares (Bi-ALS) algorithm is used to jointly estimate the MSMKron product S and the channel tensor $H^{\left(SR\right)}$. Then, the KronF algorithm is applied to separate the symbol matrices. The Bi-ALS algorithm results from the minimization of the following cost function deduced from Equation (16):

(27) $$\min_{S,H^{\left(SR\right)}}{\left\|X_{c}^{\left(SR\right)}-G_{c}^{\left(S\right)}\times_{1}H^{\left(SR\right)}\times_{2}S\times_{3}I_{F}\times_{4}I_{P}\right\|}_{F}^{2},$$

where ${\left\|\cdot \right\|}_{F}$ is the Frobenius norm. The Bi-ALS method replaces the optimization problem (27) by two LS sub-problems deduced from the matrix unfoldings (17) and (18), leading to the alternate minimization of the following LS criteria:

(28) $$\min_{H_{FM_{S}\times M_{R}}^{\left(SR\right)}}{\left\|X_{FPN\times M_{R}}^{\left(SR\right)}-\left[\left(I_{FP}⊗\hat{S}\left[it-1\right]\right)G_{FPR\times FM_{S}}^{\left(S\right)}\right]H_{FM_{S}\times M_{R}}^{\left(SR\right)}\right\|}_{F}^{2}⟶{\hat{H}}_{FM_{S}\times M_{R}}^{\left(SR\right)}\left[it\right],$$

(29) $$\min_{S}{\left\|X_{PFM_{R}\times N}^{\left(SR\right)}-\left(I_{P}⊗\mathrm{bdiag}\left({\hat{H}}_{..f}^{\left(SR\right)}\left[it\right]\right)\right)G_{PFM_{S}\times R}^{\left(S\right)}S^{T}\right\|}_{F}^{2}⟶{\hat{S}}^{T}\left[it\right].$$

The update equations at iteration [it] are given by:

(30) $${\hat{H}}_{FM_{S}\times M_{R}}^{\left(SR\right)}\left[it\right]={\left[\left(I_{FP}⊗\hat{S}\left[it-1\right]\right)G_{FPR\times FM_{S}}^{\left(S\right)}\right]}^{†}X_{FPN\times M_{R}}^{\left(SR\right)},$$

(31) $${\hat{S}}^{T}\left[it\right]={\left[\left(I_{P}⊗\mathrm{bdiag}\left({\hat{H}}_{..f}^{\left(SR\right)}\left[it\right]\right)\right)G_{PFM_{S}\times R}^{\left(S\right)}\right]}^{†}X_{PFM_{R}\times N}^{\left(SR\right)}.$$

The matrices $\left[\left(I_{FP}⊗S\right)G_{FPR\times FM_{S}}^{\left(S\right)}\right]$ and $\left[\left(I_{P}⊗\mathrm{bdiag}\left(H_{..f}^{\left(SR\right)}\right)\right)G_{PFM_{S}\times R}^{\left(S\right)}\right]$ must have full column rank to ensure the uniqueness of the LS estimates, which implies the following necessary conditions: $M_{S}\leq PN$ and $R\leq PFM_{R}$.

To simplify the computation of the estimate ${\hat{H}}_{FM_{S}\times M_{R}}^{\left(SR\right)}$ in Equation (30), we assume that the matrices ${\left[G_{PR\times M_{S}}^{\left(S\right)}\right]}_{f}$ and S have full column rank, which implies: $M_{S}\leq PR$ and R≤N, respectively. Moreover, to simplify the computation of $\hat{S}$ in Equation (31), we assume that the unfolding $G_{PFM_{S}\times R}^{\left(S\right)}$ is chosen as a full column rank truncated DFT matrix, which allows us to replace its pseudo-inverse by its transconjugate, implying the necessary condition: $R\leq PFM_{S}$. We also assume that $H_{..f}^{\left(SR\right)}$ has full column rank, implying $M_{S}\leq M_{R}$. Exploiting these assumptions and substituting the unfolding $G_{FPR\times FM_{S}}^{\left(S\right)}$ by Equation (20) simplifies the LS estimates (30) and (31) as:

(32) $${\hat{H}}_{FM_{S}\times M_{R}}^{\left(SR\right)}\left[it\right]=\mathrm{bdiag}\left({\left[G_{PR\times M_{S}}^{\left(S\right)}\right]}_{f}^{†}\right)\left(I_{FP}⊗{\hat{S}}^{†}\left[it-1\right]\right)X_{FPN\times M_{R}}^{\left(SR\right)},$$

(33) $${\hat{S}}^{T}\left[it\right]={\left(G_{PFM_{S}\times R}^{\left(S\right)}\right)}^{H}\left(I_{P}⊗\mathrm{bdiag}\left({\hat{H}}_{..f}^{(SR)†}\left[it\right]\right)\right)X_{PFM_{R}\times N}^{\left(SR\right)}.$$

The Bi-ALS algorithms (32) and (33) are simplified versions of (30) and (31) in terms of pseudo-inverses computation at the price of additional constraints on the design parameters.

The error at the ${\left[it\right]}^{th}$ iteration, deduced from (17), is considered for deciding the convergence of the Bi-ALS algorithm:

(34) $$err\left[it\right]=\|X_{FPN\times M_{R}}^{\left(SR\right)}-\left(I_{FP}⊗\hat{S}\left[it\right]\right)G_{FPR\times FM_{S}}^{\left(S\right)}{\hat{H}}_{FM_{S}\times M_{R}}^{\left(SR\right)}{\left[it\right]\|}_{F}^{2}.$$

Convergence at the ${\left[it\right]}^{th}$ iteration is declared when this error does not significantly change between two successive iterations, i.e., |err[it−1]−err[it]|≤ϵ, where ϵ is a predefined threshold. Since the core tensor $G^{\left(S\right)}$ is assumed to be known, there is no permutation ambiguity, and the generalized Tucker model (16) is unique up to scalar scaling ambiguities. The LS estimates ${\hat{\hat{H}}}_{FM_{S}\times M_{R}}^{\left(SR\right)}$ and $\hat{\hat{S}}$, at convergence, after correcting the ambiguities are given by:

(35) $$\begin{array}{cc} \hat{\hat{S}}=\hat{S}{\left(\lambda^{\left(S\right)}\right)}^{-1}, & {\hat{\hat{H}}}_{FM_{S}\times M_{R}}^{\left(SR\right)}={\hat{H}}_{FM_{S}\times M_{R}}^{\left(SR\right)}{\left(\lambda^{\left(H\right)}\right)}^{-1},\;\;\mathrm{with}\;\;\lambda^{\left(S\right)}\lambda^{\left(H\right)}=1. \end{array}$$

For eliminating these scaling ambiguities, it is sufficient to assume that one element of S is known a priori, e.g., $s_{11}=1$. Under this assumption, $\lambda^{\left(S\right)}$ is calculated as: $\lambda^{\left(S\right)}={\hat{s}}_{11}$. The symbol matrices $S^{\left(l\right)}$ are then estimated by means of the KronF algorithm presented in Appendix A, minimizing the following LS cost function:

(36) $$\min_{S^{\left(l\right)},l\in \left[1,L\right]}{\left\|\hat{\hat{S}}-S^{\left(1\right)}⊗...⊗S^{\left(L\right)}\right\|}_{F}^{2}.$$

After applying the KronF algorithm, the estimated symbol matrix ${\hat{S}}^{\left(l\right)}$ is obtained by unvectorizing ${\hat{s}}^{\left(l\right)}$ as:

(37) $${\hat{S}}^{\left(l\right)}=\mathrm{unvec}\left({\hat{s}}^{\left(l\right)}\right)\in C^{N_{l}\times R_{l}},$$

and assuming $s_{11}^{\left(l\right)}=1$, the scalar ambiguity is corrected by:

(38) $${\hat{\hat{S}}}^{\left(l\right)}={\hat{S}}^{\left(l\right)}{\left({\hat{s}}_{11}^{\left(l\right)}\right)}^{-1}.$$

As mentioned previously, the Bi-ALS-KronF receiver at the destination can be deduced from the one at the relay, using the correspondences (23) and (24), to estimate the channel $H^{\left(RD\right)}\in C^{M_{D}\times M_{R}\times F}$ and the symbol matrices denoted $S_{R}^{\left(l\right)}\in C^{N_{l}\times R_{l}}$. To eliminate the scaling ambiguities in the second hop, we use the same relation (38) for the KronF algorithm. At each hop, the estimated symbols are obtained after a projection onto the symbol alphabet. The Bi-ALS-KronF algorithm is summarized in Algorithm 1.
**Algorithm 1** Bi-ALS-KronF Receiver for Estimating the Symbol Matrices $S^{\left(l\right)}$ and the Channels $H^{\left(SR\right)}$ and $H^{\left(RD\right)}$.**Input:** tensors $X^{\left(SR\right)}$, $X^{\left(RD\right)}$, $G^{\left(S\right)}$, $G^{\left(R\right)}$**Output:** Estimated symbol matrices and channels**First hop: source–relay****- Step 1: Bi-ALS algorithm**it=0(1) Initialization of $S^{\left(l\right)}\left[0\right]$ with symbols randomly drawn from the alphabet and $s_{11}^{\left(l\right)}=1$, for l∈[1,L].(2) Update the estimates of $H_{FM_{S}\times M_{R}}^{\left(SR\right)}$ and S using Equations (30) and (31) or (32) and (33).(3) Calculate the error (34) and |err[it−1]−err[it]|.- **if** |err[it−1]−err[it]|≤ϵ or it= maximum number of iterations- **stop**- **else** it→it+1;(4) Eliminate the scaling ambiguities using Equation (35). **- Step 2: KronF algorithm**(5) Build the rank-one tensor: $\hat{\hat{S}}=\mathrm{reshape}\left(\hat{\hat{S}},\left[R_{1}N_{1},...,R_{L}N_{L}\right]\right)$.(6) Estimate each vector ${\hat{s}}^{\left(l\right)}$ by means of the KronF algorithm recalled in Appendix A, and unvectorize it using Equation (37).(7) Eliminate the scaling ambiguities using Equation (38).(8) Project the estimated symbols onto the symbol alphabet. **Second hop: relay–destination****- Step 1: Bi-ALS algorithm**- Apply the stages (1) to (4) of the first hop, using the correspondences (23) and (24). **- Step 2: KronF algorithm**- Apply the stages (5) to (8) of the first hop, using the correspondences (23) and (24). 

### 4.2. THOSVD-Based Receiver

The THOSVD-based receiver is proposed to jointly estimate the channels and the symbol matrices. This closed-form solution can be viewed as a generalization of the KronF algorithm used to separate the symbol matrices. The difference is that we can now simultaneously estimate all the matrices ($H_{M_{R}\times FM_{S}}^{\left(SR\right)},S^{\left(1\right)},\ldots ,S^{\left(L\right)}$). From the matrix unfolding (19), with S and $G_{FM_{S}R\times FP}^{\left(S\right)}$ replaced by their expressions (8) and (21), we deduce the following LS estimate of the multiple Kronecker product:

(39) $$Z^{\left(SR\right)}≜\hat{H_{M_{R}\times FM_{S}}^{\left(SR\right)}⊗S^{\left(1\right)}⊗\ldots ⊗S^{\left(L\right)}}=X_{M_{R}N\times FP}^{\left(SR\right)}\left[\mathrm{bdiag}\left({\left[G_{M_{S}R\times P}^{\left(S\right)}\right]}_{f}^{†}\right)\right],$$

with $Z^{\left(SR\right)}\in C^{M_{R}N\times FM_{S}R}$. The unfolding ${\left[G_{M_{S}R\times P}^{\left(S\right)}\right]}_{f}$ must be full row rank for ensuring the uniqueness of this LS estimate, which induces the necessary condition: $M_{S}R\leq P$. The matrices $S^{\left(l\right)}$ and $H_{M_{R}\times FM_{S}}^{\left(SR\right)}$ are jointly estimated by means of the rank-one approximation-based KronF algorithm, which is described in Appendix A. The THOSVD receiver at the destination is deduced from the one at the relay, using the correspondences (23) and (24), to estimate the channel $H^{\left(RD\right)}$ and the symbol matrices $S_{R}^{\left(l\right)}$. The THOSVD receiver is summarized in Algorithm 2.
**Algorithm 2** THOSVD Receiver for Estimating the Symbol Matrices $S^{\left(l\right)}$ and the Channels $H^{\left(SR\right)}$ and $H^{\left(RD\right)}$.**Input:** tensors $X^{\left(SR\right)}$, $X^{\left(RD\right)}$, $G^{\left(S\right)}$, $G^{\left(R\right)}$**Output:** Estimated symbol matrices and channels**First hop: source–relay**(1) Calculate the LS estimate $Z^{\left(SR\right)}$ defined in (39).(2) Build the rank-one tensor $Z^{\left(SR\right)}$ of size $R_{1}N_{1}\times \ldots \times R_{L}N_{L}\times FM_{S}M_{R}$ from $Z^{\left(SR\right)}$.(3) Compute the SVD of each mode-*n* unfolding of $Z^{\left(SR\right)}$, and calculate the estimates ${\hat{s}}^{\left(l\right)}=\mathrm{vec}\left({\hat{S}}^{\left(l\right)}\right)$ and ${\hat{h}}^{\left(SR\right)}=\mathrm{vec}\left({\hat{H}}_{M_{R}\times FM_{S}}^{\left(SR\right)}\right)$ as the first left singular vector of each mode-*n* unfolding.(4) Unvectorize ${\hat{s}}^{\left(l\right)}$ and ${\hat{h}}^{\left(SR\right)}$ to obtain the estimates ${\hat{\hat{S}}}^{\left(l\right)}$ and ${\hat{\hat{H}}}_{M_{R}\times FM_{S}}^{\left(SR\right)}$.(5) Eliminate the scaling ambiguities.(6) Project the estimated symbols onto the symbol alphabet. **Second hop: relay–destination**- Apply the stages (1) to (6) of the first hop, using the correspondences (23) and (24).

### 4.3. Zero-Forcing (ZF)-KronF Receiver

To evaluate the impact of the design parameters on the system performance, we use the zero-forcing (ZF)-KronF receiver, which assumes a perfect channel knowledge. The LS estimate of S is obtained using (31) or (33), with $H_{..f}^{\left(SR\right)}\left[it\right]$ replaced by the true channel slice $H_{..f}^{\left(SR\right)}$, which gives:

(40) $${\hat{S}}_{ZF}^{T}={\left[\left(I_{P}⊗\mathrm{bdiag}\left(H_{..f}^{\left(SR\right)}\right)\right)G_{PFM_{S}\times R}^{\left(S\right)}\right]}^{†}X_{PFM_{R}\times N}^{\left(SR\right)},$$

or

(41) $${\hat{S}}_{ZF}^{T}={\left(G_{PFM_{S}\times R}^{\left(S\right)}\right)}^{H}\left(I_{P}⊗\mathrm{bdiag}\left(H_{..f}^{(SR)†}\right)\right)X_{PFM_{R}\times N}^{\left(SR\right)}.$$

As for the Bi-ALS algorithm, the use of (40) or (41) implies the following necessary conditions: $R\leq PFM_{R}$ or $R\leq PFM_{S}$, and $M_{S}\leq M_{R}$. Then, the symbol matrices $S^{\left(l\right)}$ are estimated using the KronF algorithm as in the second step of the Bi-ALS-KronF receiver. For the second hop, the ZF-KronF receiver is similar to the one in the first hop with the correspondences (23) and (24), $H_{..f}^{\left(RD\right)}$ considered known and the matrix unfolding $G_{PFM_{R}\times R}^{\left(R\right)}$ chosen as a truncated DFT matrix. The uniqueness of the ZF solution for the second hop implies the necessary conditions: $R\leq PFM_{D}$ or $R\leq PFM_{R}$, and $M_{R}\leq M_{D}$.

Table 4 summarizes the necessary conditions for parameter identifiability with each receiver. Comparing the identifiability conditions for the Bi-ALS-KronF algorithms (32) and (33) with the ones for the Bi-ALS-KronF algorithms (30) and (31), we can deduce some implications. Indeed, for the estimate (32), the conditions $M_{S}\leq PR$ and R≤N imply $M_{S}\leq PN$, i.e., the identifiability condition for the LS solution (30). For the estimate (33), the conditions $R\leq PFM_{S}$ and $M_{S}\leq M_{R}$ imply $R\leq PFM_{R}$, i.e., the identifiability condition for the LS solution (31). In other words, if the identifiability conditions for (32) and (33) are satisfied, then the ones for the Bi-ALS algorithm (30) and (31) are automatically satisfied. Note also that $R\leq PFM_{S}$ and $M_{S}\leq PR$ imply $R\leq P^{2}FR$, which is always satisfied. Therefore, the condition $M_{S}\leq PR$ can be discarded. We can also conclude that the THOSVD receiver is more restrictive than the Bi-ALS receivers in the sense that a higher value of *P* is required, implying a reduction in the transmission rate. As the ZF-KronF receiver (41) only estimates the symbol matrices, its identifiability conditions are a subset of those of the second Bi-ALS-KronF receiver.

## 5. Computational Complexity

In this section, we compare the computational complexity of the proposed THOSVD and Bi-ALS-KronF receivers by evaluating the cost of SVD calculation, which is the most expensive matrix operation. Note that for a matrix of dimensions I×J, the complexity of SVD computation is *O*$\left(IJ\;\min(I,J)\right)$. The complexities are evaluated by taking the identifiability conditions into account.

The computational complexity of the HOSVD algorithm for an *N*-th-order tensor $X\in R^{I_{1}\times \cdots \times I_{N}}$ is of the order of $O\left(\left(\sum_{n=1}^{N}I_{n}\right)\prod_{q=1}^{N}I_{q}\right)$ if $I_{n}\leq \prod_{q\neq n}^{N}I_{q}$, requiring to compute *N* SVDs of $I_{n}\times I_{n+1}\ldots I_{N}I_{1}\ldots I_{n-1}$ matrices for n∈[1,N].

The ALS algorithm requires, at each iteration, the overall computational complexity $O\left(R^{2}\sum_{n=1}^{N}\left(\prod_{q\neq n}^{N}I_{q}\right)\right)$ to compute the PARAFAC decomposition of a tensor $X\in R^{I_{1}\times \cdots \times I_{N}}$ assumed to be of rank *R*. This algorithm requires calculating *N* LS estimates, which needs to pseudo-inverse $\prod_{q\neq n}^{N}I_{q}\times R$ matrices, for n∈[1,N].

For estimating the *L* symbol matrices from their Kronecker product, the KronF algorithm has a complexity of $O(\left(\sum_{l=1}^{L}N_{l}R_{l}\right)$$\prod_{q=1}^{L}N_{q}R_{q})$ flops.

In Table 5, the computational complexities of the Bi-ALS-KronF and THOSVD receivers are compared for the first hop. The computational complexities for the second hop can be easily derived using the correspondences (24) between the dimensions.

Note that simplifying the pseudo-inverses in (30) and (31) results in less computational complexity for the Bi-ALS-KronF (32) and (33) than for Bi-ALS-KronF (30) and (31). Regarding the computational complexity of the closed and form THOSVD and based receiver, it is generally lower than the one of the iterative Bi-ALS algorithms, which depends on the number of iterations needed for convergence.

## 6. Simulation Results

In this section, we evaluate the performance of the proposed two-hop OFDM-CDMA MIMO system and the associated receivers. First, in Section 6.1, we describe the simulations and present the considered performance criteria. In Section 6.2, we study the impact of design parameters on the symbol error rate (SER), using the ZF-KronF receiver. Finally, in Section 6.3, the proposed semi-blind receivers are compared in terms of SER and channel normalized mean square error (NMSE).

### 6.1. Description of the Simulations

The noisy signals received at each hop, $Y^{\left(SR\right)}$ and $Y^{\left(RD\right)}$, respectively, are simulated as:

(42) $$Y^{\left(SR\right)}=X^{\left(SR\right)}+\alpha^{\left(SR\right)}N^{\left(SR\right)}\in C^{M_{R}\times N_{1}\times ...\times N_{L}\times F\times P},$$

(43) $$Y^{\left(RD\right)}=X^{\left(RD\right)}+\alpha^{\left(RD\right)}N^{\left(RD\right)}\in C^{M_{D}\times N_{1}\times ...\times N_{L}\times F\times P},$$

where $N^{\left(SR\right)}\in C^{M_{R}\times N_{1}\times ...\times N_{L}\times F\times P}$ and $N^{\left(RD\right)}\in C^{M_{D}\times N_{1}\times ...\times N_{L}\times F\times P}$ are additive white Gaussian noise (AWGN) tensors whose entries are zero-mean circularly symmetric complex-valued Gaussian random variables, the tensors $X^{\left(SR\right)}$ and $X^{\left(RD\right)}$ contain the noise-free received signals obtained by means of Equations (15) and (26), respectively, and $\alpha^{\left(SR\right)}$ and $\alpha^{\left(RD\right)}$ allow fixing the signal-to-noise ratio (SNR) calculated as:

(44) $${\mathrm{SNR}}^{\left(SR\right)}=20\log\left(\frac{\|X^{\left(SR\right)}{\|}_{F}}{\alpha^{\left(SR\right)}{\left\|N^{\left(SR\right)}\right\|}_{F}}\right),$$

(45) $${\mathrm{SNR}}^{\left(RD\right)}=20\log\left(\frac{\|X^{\left(RD\right)}{\|}_{F}}{\alpha^{\left(RD\right)}{\left\|N^{\left(RD\right)}\right\|}_{F}}\right),$$

which gives $\alpha^{\left(SR\right)}=\frac{\|X^{\left(SR\right)}{\|}_{F}}{\|N^{\left(SR\right)}{\|}_{F}}10^{-\mathrm{SNR}/20}$ and $\alpha^{\left(RD\right)}=\frac{\|X^{\left(RD\right)}{\|}_{F}}{\|N^{\left(RD\right)}{\|}_{F}}10^{-\mathrm{SNR}/20}$. Note that the SNRs at the relay and destination nodes are chosen equal in the simulations. The channel tensors $H^{\left(SR\right)}$ and $H^{\left(RD\right)}$ have i.i.d. complex Gaussian entries. The symbols of symbol matrices $S^{\left(l\right)}$, for l∈[1,L], are randomly generated from the 16-QAM (Quadrature Amplitude Modulation) alphabet with a uniform distribution. It is worth mentioning that our proposed coding scheme and semi-blind receivers are not dependent on a specific choice for the modulation format as presented in [34,35]. The proposed system may operate with any modulation, although the resulting SER performance and transmission rate will be affected by this choice. For instance, increasing the modulation cardinality of M-PSK (phase-shift keying) or M-QAM type constellations (under the same total transmit power constraint) would result in a higher transmission rate at the cost of an SER performance degradation. In this work, we have adopted 16-QAM since it offers a good tradeoff between SER performance and transmission rate

As mentioned before, the coding tensors are designed for each Monte Carlo run: in such a way that, their matrix unfoldings $G_{PFM_{S}\times R}^{\left(S\right)}$ and $G_{PFM_{R}\times R}^{\left(R\right)}$ are truncated DFT matrices. The performance criteria, plotted versus SNR, are calculated as:

(46) $$\mathrm{NMSE}\left(Z\right)=\frac{1}{K}\sum_{k=1}^{K}\frac{\|{\hat{Z}}_{k}-Z_{k}{\|}_{F}^{2}}{\|Z_{k}{\|}_{F}^{2}},$$

where ${\hat{Z}}_{k}$ is the tensor $Z_{k}$ estimated at the $k^{th}$ run, with $Z_{k}\in \left\{H_{k}^{\left(SR\right)},H_{k}^{\left(RD\right)}\right\}$. The SER and NMSE are calculated by averaging the results over $K=5.10^{4}$ Monte Carlo runs, after truncating the 5% worse and 5% better values to eliminate the influence of ill-convergence and outliers.

The transmission rate *T* is given by:

(47) $$T=\frac{\sum_{l=1}^{L}N_{l}R_{l}-L}{FP\prod_{l=1}^{L}N_{l}}\log_{2}\left(\mu \right),$$

where $\sum_{l=1}^{L}N_{l}R_{l}$ corresponds to the total number of transmitted symbols, *L* is the number of symbols assumed to be a priori known for ambiguity suppression, and μ denotes the number of constellation points. Note that increasing the number $N_{l}$ of symbols in the symbol matrix $S^{\left(l\right)}$ induces an increase of coding diversity and a lower transmission rate *T*, while an increase of the number *P* of repetitions implies a decrease of *T*.

### 6.2. Impact of Design Parameters

In this section, we evaluate the SER performance of the proposed system under perfect channel knowledge. In this case, we use the ZF-KronF receiver to estimate the transmitted symbol matrices by means of Equation (41). The results presented in Figures 4–9 were obtained for both hops, but due to lack of place, some SER results are shown only for the relay. All parameters used for the simulations are provided in Table 6. Note that the default values of these parameters are chosen equal to two. The corresponding transmission rates are given in Table 7.

**Table 6:** Parameters for the simulations.

Figures	Impact of	Parameters
Figure 3	**Number of symbols per data stream**	$\left(M_{S},M_{R},M_{D}\right)=\left(2,4,6\right)$; F=2;P=2;
		$R_{1}=R_{2}=2$; $N_{1}=N_{2}\in \left\{8,12,16\right\}$
Figure 4	**Number of data streams**	$\left(M_{S},M_{R},M_{D}\right)=\left(2,4,6\right)$; F=4;
		P=12; $N_{1}=N_{2}=4$; $R_{1}=R_{2}\in \left\{4,6,8\right\}$
Figure 5	**Different configurations for $N_{1}$ and $N_{2}$**	$\left(M_{S},M_{R},M_{D}\right)=\left(2,4,6\right)$; P=F=2;
		$R_{1}=R_{2}=2$; $N_{1}=4$; $N_{2}=12$
Figure 6	**Different configurations for (F, P)**	$\left(M_{S},M_{R},M_{D}\right)=\left(2,4,6\right)$; $N_{1}=N_{2}=4$; $R_{1}=R_{2}=2$;
		(F, P) ∈{(2,2),(4,2),(8,2),(2,4),(2,8)}
Figure 7	**Number of symbol matrices**	L=2: $N_{1}=N_{2}=4$; $R_{1}=R_{2}=4$; F=8;
		P=12; $\left(M_{S},M_{R},M_{D}\right)=\left(2,4,6\right)$
		L=3: $N_{1}=N_{2}=4$;
		$N_{3}=1$; $R_{1}=4$; $R_{2}=2$; $R_{3}=9$;
		F=8; P=12; $\left(M_{S},M_{R},M_{D}\right)=\left(8,8,9\right)$
		L=5: $N_{1}=N_{2}=N_{3}=N_{4}=2$; $N_{5}=1$; $R_{1}=R_{2}=R_{3}=R_{4}=4$; $R_{5}=3$; F=8; P=12; $\left(M_{S},M_{R},M_{D}\right)=\left(8,8,9\right)$
Figure 8	**Different antenna configurations**	$N_{1}=N_{2}=4$; $R_{1}=R_{2}=2$; F=2; P=4;
		$\left(M_{S},M_{R},M_{D}\right)\in \left\{\left(2,4,6\right),\left(4,2,6\right),\left(2,2,4\right),\left(2,6,6\right)\right\}$
Figure 9	**Comparison of the TSTF-MSMKron and TSTF codings**	$\left(M_{S},M_{R},M_{D}\right)=\left(2,4,6\right)$; $N_{1}=N_{2}=2$; $R_{1}=3$;
		$R_{2}=4$; F=2; P=4; N=2; R=7
Figure 10, Figure 11 and Figure 12	**Comparison of the proposed semi-blind receivers**	$\left(M_{S},M_{R},M_{D}\right)=\left(2,4,4\right)$; $N_{1}=N_{2}=4$;
		$R_{1}=R_{2}=2$; P=18; F=2

**Table 7:** Transmission rate for different configurations.

Figures	Parameters	Transmission Rate (*T*)
Figure 3	$N_{1}=N_{2}\in \left\{8,12,16\right\}$	T = 0.468; 0.319; 0.242
Figure 4	$R_{1}=R_{2}\in \left\{4,6,8\right\}$	T = 0.156; 0.239; 0.322
Figure 5	$N_{1}=4$; $N_{2}=12$	T = 0.625
Figure 6	(F,P)∈{(2,2),(4,2),(8,2),(2,4),(2,8)}	T = 0.875; 0.437; 0.218; 0.437; 0.218
Figure 7	L∈{2,3,5}	T = 0.0781
Figure 8	$\left(M_{S},M_{R},M_{D}\right)\in \left\{\left(2,4,6\right),\left(4,2,6\right),\left(2,2,4\right),\left(2,6,6\right)\right\}$	T = 0.437
Figure 9	Comparison of the TSTF-MSMKron and TSTF codings	T = 1.5; $T_{S}$ = 7
Figure 10, Figure 11 and Figure 12	Comparison of the proposed semi-blind receivers	T = 0.094

**Figure 3:**
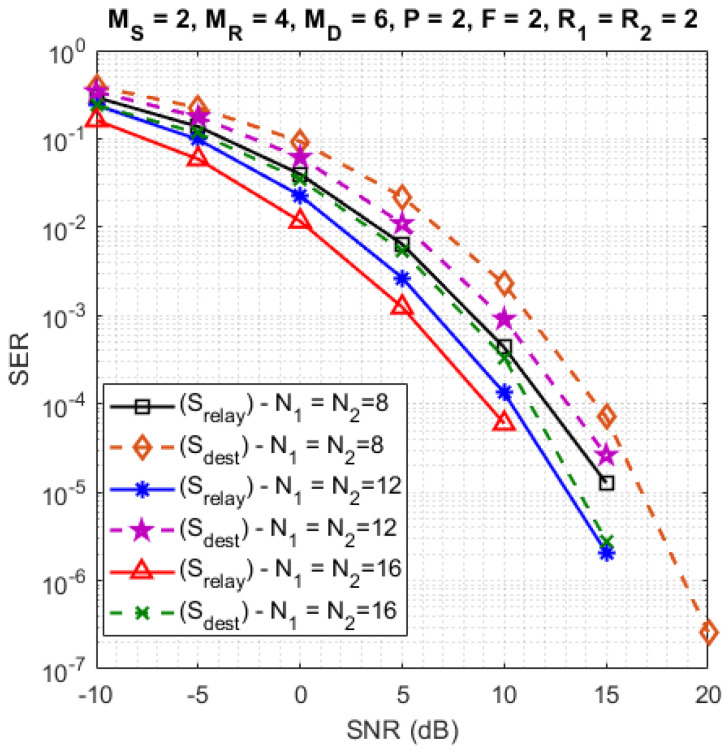
Impact of numbers of symbols per data stream.

Figure 3 shows the impact on the SER for different numbers of symbols per data stream: $N_{1}=N_{2}\in \left\{8,12,16\right\}$, where $S_{relay}$ and $S_{dest}$ denote the SER at the relay and the destination, respectively. From these simulation results, it can be concluded that the SER is improved when the numbers of symbols increase, which implies an increase of coding diversity, since $N=N_{1}N_{2}$ is a dimension of the contracted form $Y_{c}^{\left(SR\right)}$ and $Y_{c}^{\left(RD\right)}$ of the data tensors, which is not the case for $R=R_{1}R_{2}$. On the other hand, the transmission rate decreases as shown in Table 7. In addition, note that the SER at the relay is better than the one at the destination. This happens because with the DF protocol, the symbols are estimated and decoded before they are retransmitted by the relay to the destination, which induces a propagation error due to the decoding.

**Figure 4:**
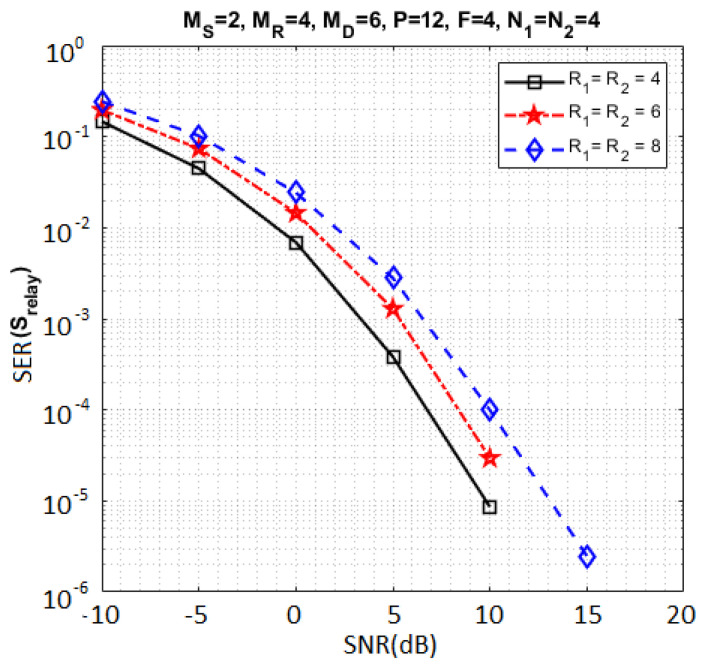
Impact of data stream numbers.

**Figure 5:**
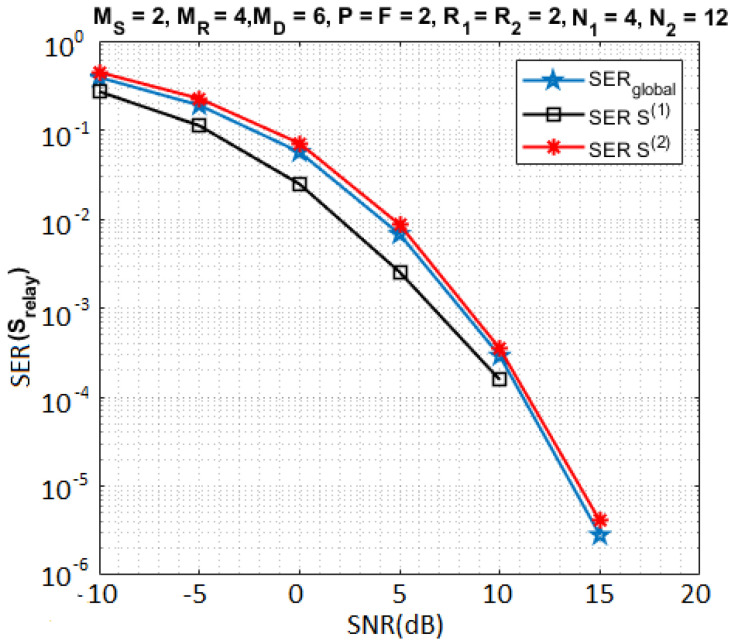
Impact on the SER of individual symbol matrices.

**Figure 6:**
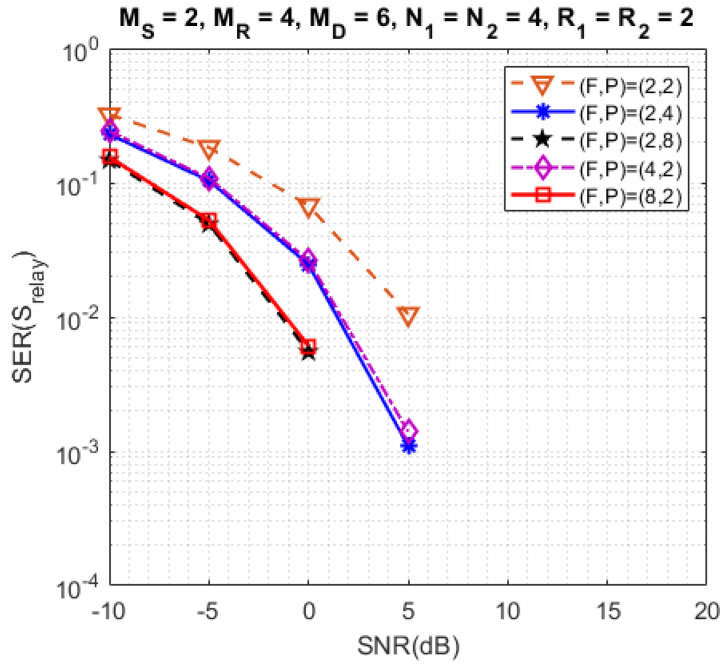
Impact of different configurations of (F,P).

**Figure 7:**
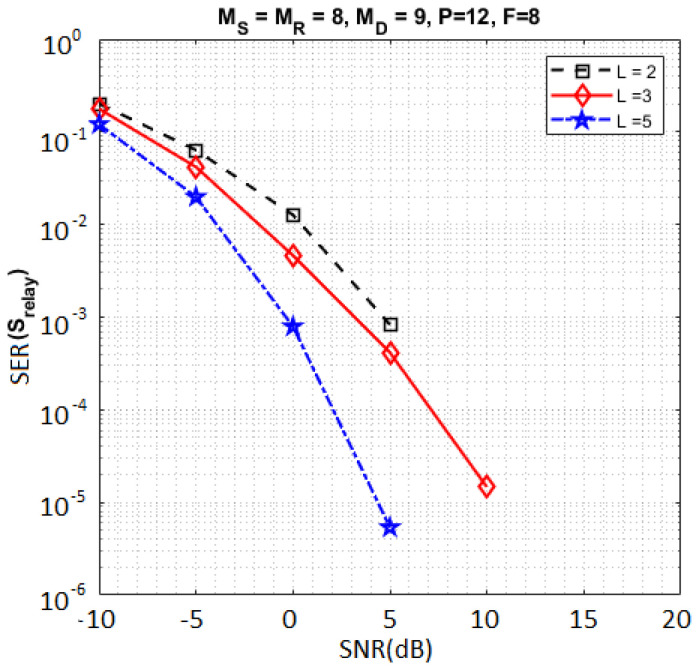
Impact of *L* on the SER.

**Figure 8:**
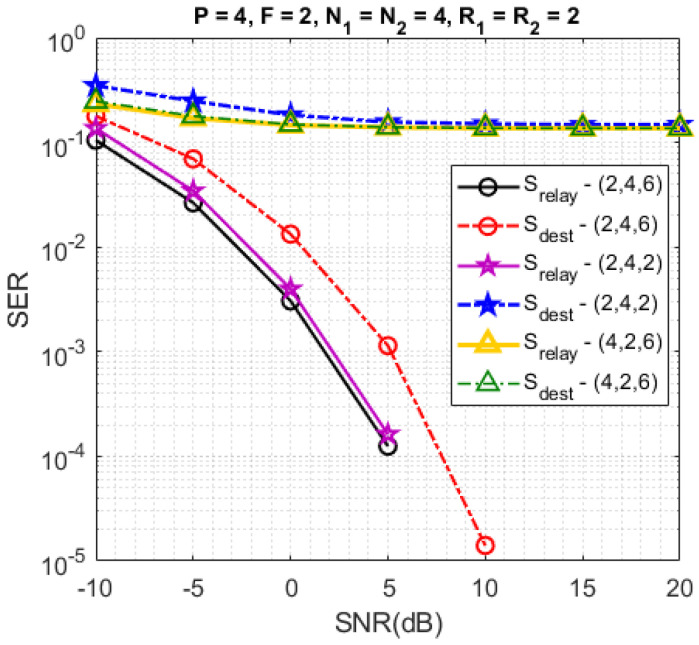
Impact of different numbers of antennas.

**Figure 9:**
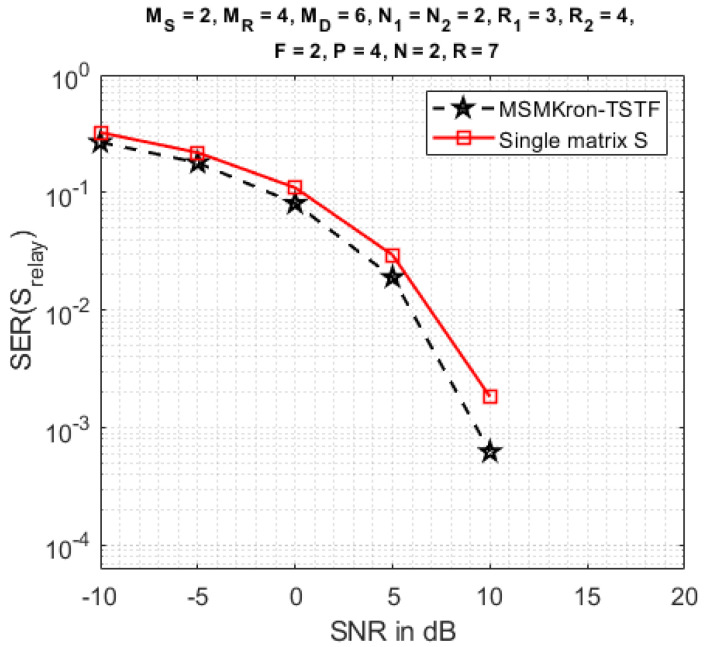
Comparison of the TSTF-MSMKron and TSTF codings.

Figure 4, Figure 5, Figure 6, Figure 7, Figure 8 and Figure 9 present the SER obtained at the relay ($S_{relay}$). Figure 4 compares the SER for three different data stream numbers: $R_{1}=R_{2}\in \left\{4,6,8\right\}$. From this figure, it can be concluded that increasing $R_{1}$ and $R_{2}$ implies an increase of the number of symbols to be estimated without increasing the number of data in the tensor $Y^{\left(SR\right)}$ for performing the symbol estimation, thus inducing a degradation of the SER, while the transmission rate increases (see Table 7).

In Figure 5, the simulation results compare the SER*_global_* with the individual SERs for $S^{\left(1\right)}$ and $S^{\left(2\right)}$ when $N_{1}=4$, $N_{2}=12$ and $R_{1}=R_{2}=2$. For this configuration, the Kronecker product between $S^{\left(1\right)}$ and $S^{\left(2\right)}$ induces a greater diversity for $S^{\left(1\right)}$ than for $S^{\left(2\right)}$ due to the fact that each symbol of $S^{\left(1\right)}$ is repeated $12R_{2}$ times while each symbol of $S^{\left(2\right)}$ is repeated only $4R_{1}$ times. That implies an SER smaller for $S^{\left(1\right)}$ than for $S^{\left(2\right)}$.

Figure 6 presents the results considering different configurations for the numbers of subcarriers (*F*) and time blocks (*P*). Note that a performance improvement is obtained when *F* and/or *P* are/is increased due to an increase of frequency and/or time diversities. On the other hand, the transmission rate decreases. We can also remark that for the same value of the product FP=8 or FP=16, the diversity gain is the same, implying very close SERs, which illustrates the symmetric role played by the frequency and time diversities in the SER performance.

In Figure 7, we compare the SER for different numbers of symbol matrices (L∈{2,3,5}). The design parameters have been chosen so that the transmission rate is the same for the three values of *L*. The MSMKron scheme with L=5 provides the best SER performance in comparison with L∈{2,3}. These results corroborate the coding gain provided by the Kronecker product of symbol matrices.

In Figure 8, the SERs are plotted for different configurations of antenna numbers $(M_{S},$$M_{R},$$M_{D})\in \left\{\left(2,4,6\right),\left(2,4,2\right),\left(4,2,6\right)\right\}$. Comparing these configurations, we note that the best SER is obtained when $M_{D}>M_{R}>M_{S}$. For the configuration (4,2,6), the SER is not good both at the relay and the destination, because the identifiability condition ($M_{S}\leq M_{R}$) at the relay is not satisfied. For the configuration (2,4,2), the SER at the relay is similar to the one for the configuration (2,4,6) because the antenna numbers $\left(M_{S},M_{R}\right)$ are the same for both configurations, but the SER at the destination is not good because the identifiability condition ($M_{R}\leq M_{D}$) at the destination is not satisfied for the configuration (2,4,2), which is not the case of the configuration (2,4,6). With this last configuration, we note that the SER at the relay is better than the one at the destination.

In Figure 9, the proposed TSTF-MSMKron coding is compared with the TSTF coding, i.e., using a single symbol matrix $S\in C^{N\times R}$ instead of a multiple Kronecker product of symbol matrices. With the TSTF coding, the symbol matrix is estimated using Equation (31), and the transmission rate is given by:

(48) $$T_{S}=\frac{R}{FP}\log_{2}\left(\mu \right).$$

For both codings, the number (14) of transmitted symbols is the same. See the design parameters in Table 6.

As expected, from Figure 9, we conclude that the TSTF-MSMKron coding gives a better SER than the TSTF coding thanks to a greater coding diversity brought by the Kronecker product of symbol matrices. As a counterpart, the transmission rate with the TSTF-MSMKron coding is smaller than the one with the TSTF coding. See Table 7.

### 6.3. Comparison of THOSVD and Bi-ALS-KronF Receivers

**Figure 10:**
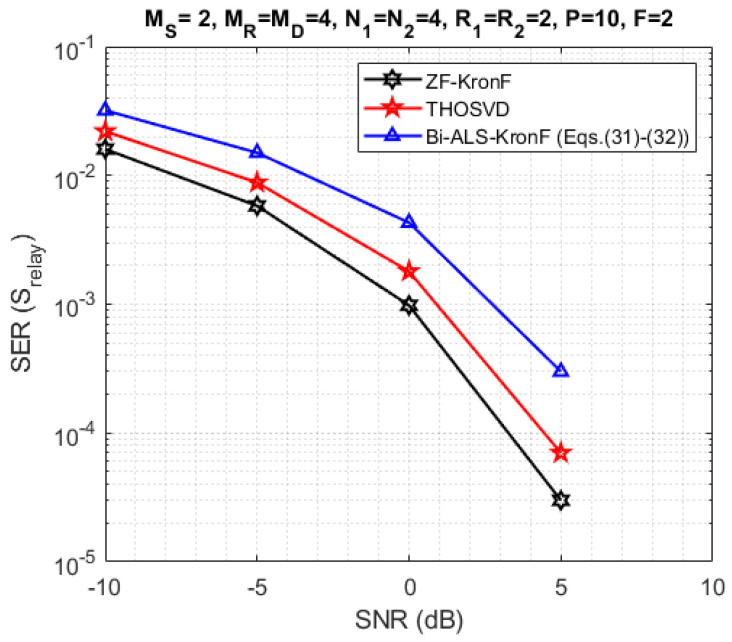
SER comparison with THOSVD, Bi-ALS-KronF Equations (32) and (33) and ZF receivers at the relay.

**Figure 11:**
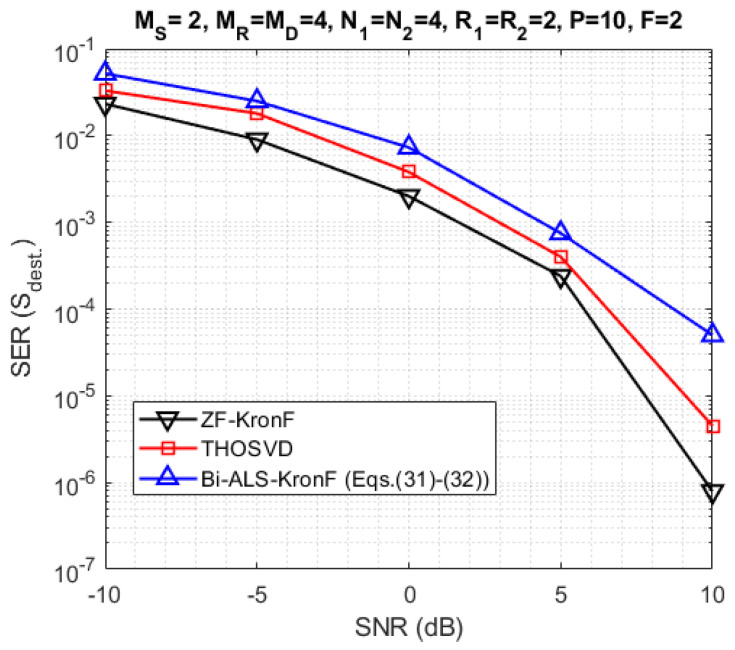
SER comparison with THOSVD, Bi-ALS-KronF Equations (32) and (33) and ZF receivers at the destination.

**Figure 12:**
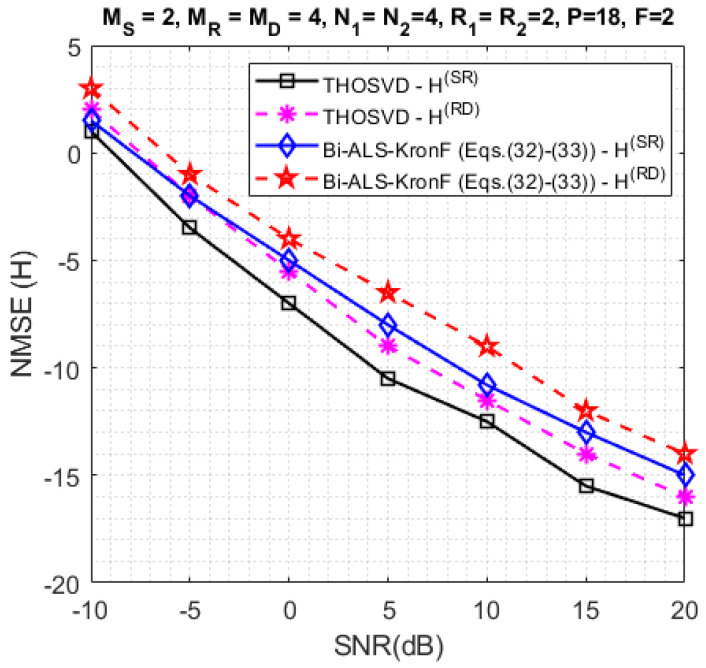
Channel NMSE comparison with THOSVD and Bi-ALS-KronF Equations (32) and (33) receivers.

In the next experiments, we compare the SERs obtained with the proposed semi-blind and ZF-KronF receivers. First, the results are presented in terms of SER at the relay ($S_{relay}$-Figure 10) and the destination ($S_{dest.}$-Figure 11). Then, we compare the performance of semi-blind receivers in terms of channel NMSE at each hop (Figure 12). For these simulations, the design parameters are fixed with the following values: $M_{S}=2$, $M_{R}=M_{D}=4$, $N_{1}=N_{2}=4$, $R_{1}=R_{2}=2$, P=18, and F=2.

From Figure 10 and Figure 11, we can conclude that the THOSVD receiver provides a better SER performance than the Bi-ALS-KronF receiver. That is due to the closed form of the THOSVD receiver allowing to jointly estimate the channel and symbol matrices, while the Bi-ALS-KronF receiver is composed of two steps, one iterative and one closed form. On the other hand, the THOSVD receiver is more constraining in terms of identifiability conditions ($M_{S}R\leq P$) than the Bi-ALS-KronF receiver, inducing a reduction of the transmission rate, as can be seen in Table 7. It can also be noted that the SER at the relay is better than the one at destination due to the error propagation caused by decoding at the relay. As expected, the ZF-KronF receiver provides the best SER due to an a priori knowledge of the channels.

In Figure 12, the channel NMSE results obtained at each hop are plotted. Note that the THOSVD receiver gives better results than the Bi-ALS-KronF one. As for the SER, this is because the THOSVD is a closed-form solution, while the Bi-ALS algorithm is iterative. Moreover, the channel estimation in the first hop is slightly better than the one in the second hop. This is due to error propagation in the re-transmission of symbol matrices after decoding at the relay.

Note that considering non-coherent receivers [36,37,38] would imply avoiding the assumption about the knowledge of the coding tensors $G^{\left(S\right)}$ and $G^{\left(R\right)}$ used at the source and the relay, which would require a fully blind approach. Such a non-coherent assumption would destroy the essential uniqueness property of the estimated channels and symbols (up to scaling ambiguities). More specifically, in the non-coherent case, the Tucker models defined in Equations (15) and (26) would be affected by rotational ambiguities, which means that the channel tensors and symbol matrices estimated at the relay and destination nodes would be linked to the true ones via a transformation by a nonsingular matrix. It should be mentioned that one possible way to ensure the successful decoding of transmitted symbols in the non-coherent case, where such rotational ambiguities are present, is to consider Grassmannian constellations for symbol matrices, as proposed in [39,40].

## 7. Conclusions

In this paper, we have proposed a new two-hop CDMA-OFDM MIMO system which combines a tensor space–time–frequency (TSTF) coding with a multiple Kronecker product of symbol matrices, leading to the so-called TSTF-MSMKron coding. This new coding makes it possible to improve the gains in diversity and throughput. We have shown that the tensors of signals received at the relay and destination nodes satisfy two generalized Tucker models whose core tensors are the coding tensors.

Assuming these coding tensors are known, two semi-blind receivers have been derived to jointly estimate the transmitted information symbols and the channels. One, called the Bi-ALS-KronF receiver, is composed of two stages. In the first stage, the iterative ALS algorithm is used to estimate the channel and the Kronecker product of symbol matrices, while in the second stage, the KronF method is applied to separate the symbol matrices. The other one, called THOSVD receiver, is a closed-form solution which allows simultaneously estimating the channel and the symbol matrices by means of SVD computations as with the KronF method. Necessary conditions for system identifiability have been established for each receiver, showing that the THOSVD receiver is more constraining than the Bi-ALS-KronF one for the choice of the number of time blocks and consequently from the data rate point of view.

It is worth mentioning that the proposed two-hop system can be easily extended to the multi-hop case owing to the use of the DF protocol at the relay, since the tensor models for the signals received at the relays and destination have the same structure (generalized Tucker models), with the correspondences (23) and (24) established between the first and second hops. These correspondences can be easily generalized to more than two hops if the same coding scheme is used at each relay.

Extensive Monte Carlo simulations have allowed illustrating the impact of all the design parameters on the SER performance using the ZF receiver. In particular, the diversity gain brought by each parameter of the TSTF-MSMKron coding has been analyzed. The performances of the proposed semi-blind receivers have been compared in terms of SER and channel NMSE. As expected, the THOSVD closed-form receiver outperforms the iterative Bi-Als-KronF receiver. Moreover, a comparison with the standard TSTF coding has corroborated the SER improvement brought by the MSMKron coding, which allows increasing the diversity gain.

Note that we have numerically evaluated the SER performance under different schemes, assuming 16-QAM constellation for all the symbol matrices involved in our MSMKron coding scheme. At this point, we do not have a theoretical SER performance evaluation. Deriving an analytical Cramer–Rao bound (CRB) for the estimated channels and symbols is challenging, and this constitutes an important perspective for this work.

Among some other perspectives of this work, we can mention an extension of the proposed relaying system to the multi-hop case using the amplify-and-forward (AF) protocol and taking resource allocation tensors into account. Such considerations will lead to new tensor models and therefore new semi-blind receivers. Other extensions concern the development of relaying systems with TSTF-MSMKron coding for double-directional dual-polarized MIMO systems and intelligent reflecting surfaces (IRS)-assisted systems.

## Figures and Tables

**Table 1:** Tensor-based MIMO cooperative systems.

Ref.	System Types	Codings	Tensor Models	Receivers
[24]	two-hop	KRST	PARAFAC/PARATUCK	ALS
[18]	two-hop	KRST	nested PARAFAC	ALS
[19]	two-hop	KRST	nested PARAFAC	KRF
[16]	two-hop	TST	nested TD	ALS-KronF
[26]	two-hop	MKRST/MKronST	nested PARAFAC	KRF/KronF
[28]	two-hop	TST	coupled nested TD	KronF
[29]	three-hop	TST + PARAFAC	nested TD	coupled SVD/ALS
[22]	three-hop	KRST	nested PARAFAC	ALS/KRF
[21]	multi-hop	TST	high-order nested TD	KronF
[25]	multi-hop	KRST	nested PARAFAC	KRF
New	two-hop	TSTF+MSMKron	generalized-Tucker	ALS-KronF/THOSVD

**Table 2:** System design parameters.

System Design Parameters	Definitions
*L*	number of symbol matrices
$R_{l}$	number of data streams in $S^{\left(l\right)}$
$N_{l}$	number of symbols in the $R_{l}^{th}$ data stream
*F*	number of subcarriers
*P*	number of time blocks
$M_{S}$	number of antennas at the source
$M_{R}$	number of antennas at the relay
$M_{D}$	number of antennas at the destination

**Table 3:** System matrices and tensors.

Symbol Matrices
$S^{\left(l\right)}\in C^{N_{l}\times R_{l}}$, for l∈[1,L]
$S=S^{\left(1\right)}⊗...⊗S^{\left(L\right)}\in C^{N\times R}$
$N=\prod_{l=1}^{L}N_{l},\;R=\prod_{l=1}^{L}R_{l}$
**Channel tensors**
$H^{\left(SR\right)}\in C^{M_{R}\times M_{S}\times F}$
$H^{\left(RD\right)}\in C^{M_{D}\times M_{R}\times F}$
**Space-time-frequency coding tensors**
$G^{\left(S\right)}\in C^{M_{S}\times R_{1}\times ...\times R_{L}\times F\times P}$
$G^{\left(R\right)}\in C^{M_{R}\times R_{1}\times ...\times R_{L}\times F\times P}$
**Received signals tensors**
$X^{\left(SR\right)}\in C^{M_{R}\times N_{1}\times ...\times N_{L}\times F\times P}$
$X^{\left(RD\right)}\in C^{M_{D}\times N_{1}\times ...\times N_{L}\times F\times P}$

**Table 4:** Identifiability conditions for the receivers.

Receiver	Identifiability Conditions (First Hop)	Identifiability Conditions (Second Hop)
Bi-ALS-KronF	$R\leq PFM_{R}$;	$R\leq PFM_{D}$;
Equations (30) and (31)	$M_{S}\leq PN$	$M_{R}\leq PN$
Bi-ALS-KronF	$M_{S}\leq \min\left(PR,M_{R}\right)$;	$M_{R}\leq \min\left(PR,M_{D}\right)$;
Equations (32) and (33)	$R\leq \min(N,PFM_{S})$	$R\leq \min(N,PFM_{R})$
THOSVD	$M_{S}R\leq P$;	$M_{R}R\leq P$
ZF-KronF (40)	$R\leq PFM_{R}$	$R\leq PFM_{D}$
ZF-KronF (41)	$R\leq PFM_{S}$; $M_{S}\leq M_{R}$	$R\leq PFM_{R}$; $M_{R}\leq M_{D}$

**Table 5:** Computational complexity of the Bi-AKS-KronF and THOSVD algorithms at the first hop.

Algorithms	Computational Complexity
Bi-ALS-KronF (30) and (31)	$O\left(F^{3}M_{S}^{2}PN\right)+O\left(R^{2}PFM_{R}\right)+O\left(\left(\sum_{l=1}^{L}N_{l}R_{l}\right)\prod_{q=1}^{L}N_{q}R_{q}\right)$
Bi-ALS-KronF (32) and (33)	$O\left(M_{S}^{2}PR\right)+O\left(R^{2}N\right)+O\left(F^{3}M_{R}^{2}M_{S}\right)+O\left(\left(\sum_{l=1}^{L}N_{l}R_{l}\right)\prod_{q=1}^{L}N_{q}R_{q}\right)$
THOSVD	$O\left(P^{2}FM_{S}R\right)+O\left(F^{2}M_{S}^{2}M_{R}\right)+O\left(FM_{S}M_{R}\left(\sum_{l=1}^{L}N_{l}R_{l}\right)\prod_{q=1}^{L}N_{q}R_{q}\right)$

## Data Availability

Not applicable.

## Abbreviations

The following abbreviations are used in this manuscript:

4G	fourth-generation
5G	fifth-generation
6G	sixth-generation
AF	amplify-and-forward
ALS	alternating least squares
Bi-ALS	bi-alternating least squares
CDMA	code division multiplexing access
CSI	channel state information
DF	decode-forward
DFT	discrete Fourier transform
DKRF	double KRF
HONTD	high-order nested Tucker decomposition
IoT	internet of things
KRF	Khatri–Rao factorization
KRST	Khatri–Rao space-time
KRSTF	Khatri–Rao space-time-frequency
KronF	Kronecker factorization
LM	Levenbergh–Marquardt
LS	least squares
LSKP	LS estimation of Kronecker products
MIMO	multiple input multiple output
MKRF	multiple Khatri–Rao factorization
MKRSM	multiple Khatri–Rao product of symbol matrices
MKRST	multiple Khatri–Rao space–time
MKronST	multiple Kronecker space–time
MSMKron	multiple symbol matrices Kronecker product
NMSE	normalized mean square error
OFDM	orthogonal frequency division multiplexing
PARAFAC	parallel factors analysis
QAM	quadrature amplitude modulation
PSK	phase-shift keying
SER	symbol error rate
SNR	signal-to-noise ratio
ST	space–time
SVD	signal value decomposition
TD	Tucker decomposition
THOSVD	truncated higher-order singular value decomposition
TSTF	tensor space–time frequency
ZF	zero-forcing

## Appendix A. Kronecker Factorization (KronF) Algorithm

In this section, the KronF algorithm is presented for estimating the matrix factors of a multiple Kronecker product $C=A^{\left(1\right)}⊗\ldots ⊗A^{\left(N\right)}\in C^{I_{1}\ldots I_{N}\times R_{1}\ldots R_{N}}$, with $A^{\left(n\right)}\in C^{I_{n}\times R_{n}}$, for n∈[1,N], by minimizing the LS cost function:

(A1) $$\min_{A^{\left(n\right)},n\in \left[1,N\right]}{\left\|C-A^{\left(1\right)}⊗\ldots ⊗A^{\left(N\right)}\right\|}_{F}^{2},$$

Following the idea introduced in [41] for a Kronecker product of two matrices, the problem is solved by rewriting the cost function (A1) in terms of approximation of a rank-one tensor built as the outer product of vectorized forms of the matrix factors, as:

(A2) $$\min_{a^{\left(n\right)},n\in \left[1,N\right]}{\left\|C-a^{\left(1\right)}\circ \ldots \circ a^{\left(N\right)}\right\|}_{F}^{2},$$

where $a^{\left(n\right)}=\mathrm{vec}\left(A^{\left(n\right)}\right)\in C^{R_{n}I_{n}}$, and $C\in C^{R_{1}I_{1}\times \ldots \times R_{N}I_{N}}$ is the rank-one tensor obtained by reshaping the multiple Kronecker product:

(A3) $$C=\mathrm{reshape}(C,\left[R_{1}I_{1},\ldots ,R_{N}I_{N}\right]).$$

Each vector $a^{\left(n\right)}$ is estimated using the THOSVD algorithm, and the matrix factor estimate ${\hat{A}}^{\left(n\right)}$ is deduced using the unvec operator [29,32,42], with a scalar ambiguity which can be eliminated assuming the knowledge of one element of $A^{\left(n\right)}$, e.g., $a_{11}^{\left(n\right)}=1$, which leads to the following corrected estimate:

(A4) $${\hat{\hat{A}}}^{\left(n\right)}={\left({\hat{a}}_{11}^{\left(n\right)}\right)}^{-1}{\hat{A}}^{\left(n\right)}.$$
